# Early clinical course after hematopoietic stem cell transplantation in children with juvenile metachromatic leukodystrophy

**DOI:** 10.1186/s40348-020-00103-7

**Published:** 2020-09-03

**Authors:** Judith Beschle, Michaela Döring, Christiane Kehrer, Christa Raabe, Ute Bayha, Manuel Strölin, Judith Böhringer, Andrea Bevot, Nadja Kaiser, Benjamin Bender, Alexander Grimm, Peter Lang, Ingo Müller, Ingeborg Krägeloh-Mann, Samuel Groeschel

**Affiliations:** 1 Department for Pediatric Neurology, University Children’s Hospital, Tübingen, Germany; 2 Department for General Pediatrics, Hematology/Oncology, University Children’s Hospital, Tübingen, Germany; 3 Center of Neurology, University Hospital, Tübingen, Germany; 4 Department of Diagnostic and Interventional Neuroradiology, Tübingen, Germany; 5 Department of Pediatric Hematology and Oncology, University Hospital Eppendorf, Hamburg, Germany

**Keywords:** Hematopoietic stem cell transplantation, Metachromatic leukodystrophy

## Abstract

**Background:**

Long-term outcomes of hematopoietic stem cell transplantation (HSCT) in children with juvenile metachromatic leukodystrophy (MLD) have been investigated systematically, while short-term effects of HSCT on the course of the disease remain to be elucidated.

**Results:**

In this study, the clinical course was evaluated over the first 24 months following HSCT, conducted at our center in 12 children with juvenile MLD (mean follow-up 6.75 years, range 3–13.5) and compared with 35 non-transplanted children with juvenile MLD. Motor function (GMFM-88 and GMFC-MLD), cognitive function (FSIQ), peripheral neuropathy (tibial nerve conduction velocity), and cerebral changes (MLD-MR severity score) were tested prospectively.

Seven children remained neurologically stable over a long period, five exhibited rapid disease progression over the first 12 to 18 months after transplantation. In the latter, time from first gross motor symptoms to loss of independent walking was significantly shorter compared with non-transplanted patients at the same stage of disease (*p* < 0.02). Positive prognostic factors were good motor function (GMFM = 100%, GMFC-MLD = 0) and a low MR severity score (≤ 17) at the time of HSCT.

**Conclusions:**

Our results show that if disease progression occurs, this happens early on after HSCT and proceeds faster than in non-transplanted children with juvenile MLD, indicating that HSCT may trigger disease progression.

## Background

Metachromatic leukodystrophy (MLD) is a rare lysosomal storage disorder caused by mutations in the gene encoding arylsulfatase A (ARSA) [1]. The disease is monogenetic and autosomal recessive. The ARSA deficiency means that degradation of galactosylceramide-3-0-sulfate (sulfatide) is no longer possible, which leads to accumulation predominantly in the central and peripheral nervous system, causing progressive demyelination and severe neurological symptoms [1].

Age at onset is variable. Two clinical forms are distinguished in children: the late-infantile (onset before 30 months) and the juvenile form (onset between 2.5 and 16 years) [1, 2]. Late-infantile MLD is characterized by a highly invariable course of disease, with rapid progression leading to complete loss of gross motor function before the age of 40 months [3]. In contrast, the disease usually progresses more slowly and is more variable in juvenile MLD. Nevertheless, once independent walking is lost, motor deterioration proceeds as rapidly as in the late-infantile form [3, 4].

Over the past 20 years, hematopoietic stem cell transplantation (HSCT) has been the only clinically available therapeutic option for juvenile MLD [5]. The rationale is that monocytic bone marrow cells can cross the blood–brain barrier, migrate into the brain, differentiate into microglia cells and produce the missing enzyme, thereby enabling re-myelination [6, 7]. It takes up to 12–24 months until disease stabilization occurs because of the slow replacement of resident tissue [5, 8–10], which in turn makes HSCT ineffective in children with the rapidly progressive late-infantile form and in juvenile patients with advanced symptoms [11]. The main reason for a good outcome in children with the juvenile form is considered to be the early clinical disease stage at transplantation, with sufficient time until severe disease progression. Patients who underwent transplantation at an early disease stage, with low motor and neurocognitive impairment, or at a pre-symptomatic stage, exhibited a better long-term outcome than patients in advanced stages of the disease [5, 6, 12–18]. Nevertheless, due to variable outcomes [8–10, 19–22] and the frequent lack of comparisons between the outcomes in transplanted and non-transplanted patients, whether HSCT is an option for treatment of MLD remains controversial [5, 6, 13, 23].

To better understand the variable long-term outcome, it is necessary to investigate the period when deterioration occurs, i.e., the first years after transplantation. Therefore, we aimed at analyzing the clinical course of the disease over the first 2 years after HSCT. In particular, we aimed to investigate whether HSCT triggers disease progression by comparing the course of the disease with non-transplanted children with juvenile MLD at the same stage of the disease. In addition, we wanted to analyze baseline parameters before HSCT for their prognostic value regarding disease stabilization.

## Subjects and methods

### Patients

Between 2001 and 2015, 12 children with juvenile MLD were transplanted at the University Children’s Hospital of Tübingen (8 females, 4 males). Table 1 summarizes patient characteristics at baseline. Mean age at HSCT was 11.6 years (range 5–18.2) and mean follow-up after transplantation was 6.75 years (range 3–13.5). Mean age at onset was 8 years (range 4.3–13.1), with three children having an early-juvenile onset (between 2.5 and 6 years of age). Some of the patients (7 out of 12) have been reported on previously with respect to long-term outcomes within a larger cohort that combined patients from three centers in Germany [12].

**Table 1:** Patient characteristics showing age of onset, baseline characertistics at HSCT, conditioning regimen, and early outcome classification

**ID**	**Age at onset** **(in years)**	**Age at HSCT + year**	**GMFC.MLD at HSCT**	**GMFM at HSCT (%)**	**FSIQ at HSCT**	**MRI-score at HSCT**	**NCV at HSCT (m/s)**	**Conditioning regimen**	**Early HSCT outcome**
**1**	13.1 (late juvenile)	13.9 2014	1	99.4	82	20	47	Treosulfan, Fludarabin	Rapidly progressive
**2**	4.3 (early juvenile)	6.0 2009	1	93.1	98	17	15	Treosulfan, Fludarabin	Stable
**3**	7.3 (late juvenile)	11.8 2015	1	99.5	79	18	22	Treosulfan, Fludarabin	Rapidly progressive
**4**	9.8 (late juvenile)	11.6 2014	0	100	77	16	41	Treosulfan, Fludarabin	Stable
**5**	4.8 (early juvenile)	4.9 2001	1	99	100	14	31	Busulfan, Cyclophosphamid	Stable
**6**	4.3 (early juvenile)	5.2 2015	1	89.6	84	19	29	Treosulfan, Fludarabin	Rapidly progressive
**7**	Pre-symptomatic	13.7 2008	0	100	112	0	34	Busulfan, Cyclophosphamid	Stable
**8**	Pre-symptomatic	15.2 2006	0	100	114	11	42	Busulfan, Cyclophosphamid	Stable
**9**	9.5 (late juvenile)	18.2 2010	0	98	68	21	50	Treosulfan, Fludarabin	Stable
**10**	Pre-symptomatic (sibling onset 9.5)	14.8 2012	0	97	91	7	53	Treosulfan, Fludarabin	Stable
**11**	11.4 (late juvenile)	13.6 2015	1	99.4	103	18	30	Treosulfan, Fludarabin	Rapidly progressive
**12**	8.1 (late juvenile)	10.1 2009	1	98.6	66	20	17	Treosulfan, Fludarabin	Rapidly progressive

Hematopoietic stem cells were derived from bone marrow (*n* = 9) or peripheral blood (*n* = 3). Nine children received stem cells from HLA-matched unrelated donors (MUD) and three children from HLA-matched family donors (MFD). Conditioning was carried out for the 9 patients transplanted since 2009 with fludarabine (5 x 30 mg/m^2^), treosulfan (3 × 14 g/m^2^), thiotepa (1 × 10 mg/kg BW). and thymoglobulin (10 mg/kg BW) or anti-thymocyte globulin (Fresenius/Neovii) (3 × 10 mg/kg BW or 3 × 20 mg/kg BW), and busulfan (4 × 3.2 mg/kg BW), cyclophosphamide (4 × 50 mg/kg BW or 2 × 60 mg/kg BW), and thymoglobulin (10 mg/kg BW) or anti-thymocyte globulin (3 × 10 mg/kg BW) in the 3 cases transplanted before 2009. Conditioning regimen was changed from Busulfan to Treosulfan due to better toxicity profile in children [24]. ARSA activity was measured in donor cells in order to ensure enzyme activity in the normal range and to avoid low activity due to heterozygosity or pseudodeficiency [1]. Transplantation-related details are given in the supplementary material.

Diagnosis of MLD was based on ARSA activity in leukocytes in combination with the excretion of sulfatides in urine. Diagnosis was confirmed by mutation analysis. Juvenile MLD was defined as “first clinical symptoms between the age of 2.5 and 16 years.” In pre-symptomatic patients, a sibling with a juvenile course or a genotype compatible with the juvenile form was used in the diagnosis, as well as either MRI changes or/and reduced nerve conduction velocity [12].

Data from 35 non-transplanted children with juvenile MLD were used as a control group. All of them (16 females, 19 males) were diagnosed at the University Children’s Hospital of Tübingen using the same criteria, mean age at onset was 6.9 years (range 2.7–15). Their clinical characteristics have been reported elsewhere [12].

Clinical data were collected within the German leukodystrophy network, Leukonet [3, 4]. The study was approved by the local Ethical Committee of the University of Tübingen (no. 401/2005). Written informed consent was given by at least one caregiver.

### Analysis of early outcomes after HSCT

Gross motor function was determined using the standardized Gross Motor Function Classification for MLD (GMFC-MLD) for transplanted as well as for non-transplanted patients. GMFC-MLD is a validated categorical scoring system with 7 levels, with 0 defining no abnormalities in gross motor function, to 6, defining loss of all gross motor function, including head control [3].

In addition, we used the GMFM-88 in transplanted patients, a test validated to prospectively evaluate children with neurological disorders [25, 26]. It consists of 88 items in 5 dimensions. Clinical examinations and videotaping of GMFM-88 were carried out by a medical doctor (C.K.) and a trained physiotherapist (C.R.).

Cognitive function was assessed using the Wechsler Intelligence Scale for Children III–V, the Wechsler Adult Intelligence Scale III–V or the Kaufman Assessment Battery for Children I–II. Examinations were carried out by one experienced psychologist (U.B.).

Motor nerve conduction velocity (NCV) of the tibial nerve was measured to assess peripheral demyelination. Normal NCV was defined as over 40 m/s [27]

The MLD MR severity score was used to quantify brain abnormalities [28]. All MRIs were scored by one experienced rater (S.G.).

These tests were conducted during regular follow-ups after HSCT over the first 5 years after transplantation: in the first 2 years, patients were examined every 6 months, and once a year from year two onwards.

Clinical outcome after HSCT was categorized into disease stabilization or progression, as done previously [12]: stable disease was defined as a loss of no more than one level in GMFC-MLD and a loss of ≤ 30 points (2 SD) in FSIQ. Progressive disease was defined accordingly.

Based on this definition for outcomes, we compared early outcome (within 24 months after HSCT) with long-term outcome (> 24 months after HSCT), to investigate whether neurological deterioration only occurs in the first years after HSCT or also thereafter.

Hematology: time to complete chimerism, transplant-related mortality and rate of complications (GvHD, rejection, VOD, sepsis, viremia, and fungal infection) were assessed (supplementary material).

### Analysis of disease progression in transplanted vs. non-transplanted patients

In order to investigate whether the rate of disease progression after HSCT differs between transplanted and non-transplanted children with juvenile MLD, we compared the time from level 1 GMFC-MLD to level 2 GMFC-MLD between transplanted and non-transplanted patients, using a survival analysis and log-rank test. Given that rapid progression in juvenile MLD is known to occur between level 2 and 5 GMFC-MLD [3], the time from level 1 to 2 was used in our study to define the phase from first abnormalities in motor function (level 1) to the beginning of rapid disease progression, which is marked by the loss of independent walking (level 2).

The analyses were regarded as explorative rather than confirmatory as the study was retrospective, with relatively small numbers. Therefore, all *P* values were considered to be descriptive. Statistical analyses were performed using SPSS.

### Prognostic values of baseline parameters for early outcome

The difference in baseline parameters between the outcome groups (as defined above) were investigated using Pearson’s chi-squared test. Baseline parameters were divided into categories based on published prognostic markers for HSCT outcomes [11; 17], such as normal/abnormal gross motor function (GMFM-88 100% vs. < 100% and GMFC-MLD 0 vs. ≥ 1), normal/abnormal FSIQ (≥ 85 vs. < 85), normal/abnormal NCV (≥ 40 m/s vs. < 40 m/s) and MR severity scores of ≤ 17 vs. > 17 [12]. In addition, age of onset and age at HSCT were tested between the outcome groups, using Mann–Whitney *U* test. In pre-symptomatic patients at HSCT, age of onset of the affected sibling was used, if available [29].

## Results

### Early outcome analysis

#### Overall survival

Overall survival was 100%. Neither transplant-related mortality occurred, nor mortality due to disease progression.

#### Gross motor function (Fig. 1a)

All patients with good gross motor function, i.e., GMFM scores near to 100% (mean 97.8%) and a GMFC-MLD level of either 0 (*n* = 5) or 1 (*n* = 7), were transplanted.

**Fig. 1:**
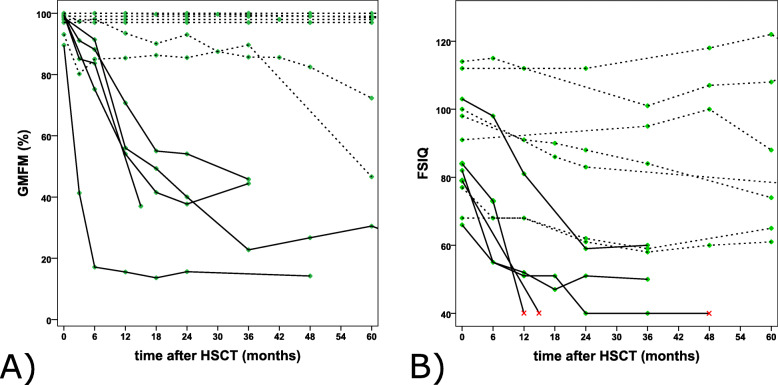
Gross motor function based on GMFM-88 (**a**) and FSIQ (**b**) is shown over time. Patients with disease stabilization are represented with dashed lines, patients with disease progression with solid lines. Patients who were no longer testable for FSIQ are shown in red

Regarding their outcome after HSCT, seven patients remained stable, e.g., exhibited no, or only mild, deterioration in motor function (≤ 1 level of GMFC-MLD). Five patients deteriorated in the first 12 to 18 months after transplantation (> 1 level of GMFC-MLD) and subsequently also remained stable, but on a lower GMFC-MLD level (level 3 (*n* = 1), 4 (*n* = 1), 5 (*n* = 3)).

#### Cognitive function (Fig. 1b)

FSIQ was variable at the time of HSCT (mean 89.25, range 66–114). Nevertheless, cognitive function did not deteriorate, or only slightly, in seven patients (≤ 2 SD, mean 8.57, range 0–20) and remained stable throughout the entire observation period. Five patients deteriorated in the first 12 to 18 months after transplantation (> 2 SD, mean 37.8, range 31–44) and subsequently also remained stable, but on a much lower level (FSIQ < 60, mean 45.8, range 40–59). Three of these five patients were no longer testable, following a deterioration in cognitive performance of FSIQ < 40.

It is important to note that patients who suffered from motor deterioration also suffered from cognitive deterioration. Therefore, if deterioration occurred in the first 12 to 18 months after HSCT, both motor and cognitive function declined.

#### NCV (Fig. 2a)

There was no relevant change in the motor NCV of the tibial nerve after HSCT. Five patients exhibited an NCV within the normal range, and seven patients exhibited pathological values. Whether abnormal or normal, these values remained relatively stable throughout the observation period. Pathological or normal values did not correlate with the deterioration in gross motor function, mentioned above.

**Fig. 2:**
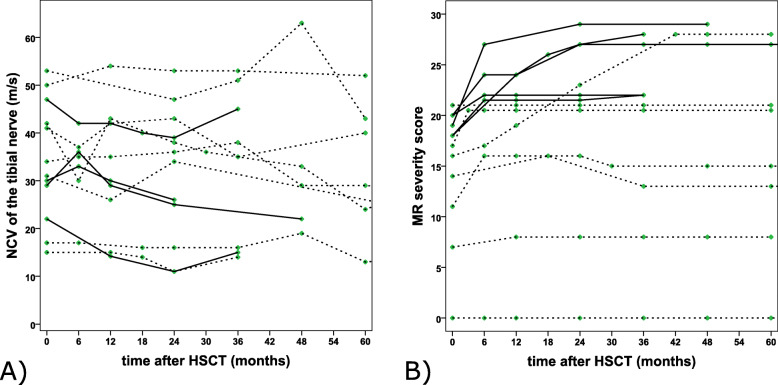
Nerve conduction velocity (NCV) of the tibial nerve (**a**) and the MRI severity score (**b**) are shown over time. Patients with disease stabilization are represented with dashed lines, patients with disease progression with solid lines

#### MR severity score (Fig. 2b)

The MR score increased in most patients over the first months after HSCT (it remained stable from baseline in two), but subsequently remained stable. This increase was most pronounced within the first 12 months, with further deterioration especially in those with higher MR severity scores up to 24 and 36 months, with stabilization thereafter. In two patients, the MRI score even slightly decreased after initial increase.

#### Early vs. long-term outcome

Over the first 2 years after HSCT, 5 of 12 patients exhibited severe disease progression in motor and cognitive function, as well as in MRI scores. Of the seven patients who remained stable 2 years after HSCT, two exhibited further disease progression during the remaining observation period. One patient progressed in GMFM to 50% 5 years after HSCT (see Fig. 1a); however, only deteriorating 1 level from GMFC-MLD 1 to 2, still remaining in the definition of stable for long-term outcome [12]. The other patient deteriorated from level 1 to 3 GMFC-MLD after 13 years (e.g., lost independent walking and was wheel chair-bound) after she suffered a lengthy period of immobilization (due to pneumonia) following orthopedic correction of a bilateral foot deformity in the context of her neuropathy, but her FSIQ remained stable.

#### Hematological outcome

All patients achieved complete chimerism (*n* = 10 after 30 days, *n* = 2 after 60 days). The rate of complications was low, with five patients suffering from grade 1 GvHD. All patients responded well to GvHD treatment. No transplant rejection occurred. Complications, such as viremia and VOD were observed in 1 case each. Sepsis/SIRS and fungal infection were not observed. There was no transplant-related mortality.

### Analysis of disease progression in transplanted vs. non-transplanted patients

**Fig. 3:**
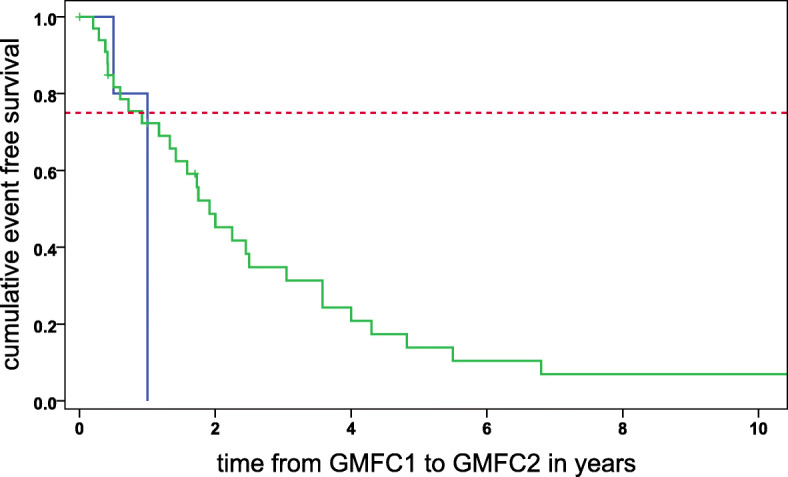
Time from GMFC-MLD1 to GMFC-MLD2 in patients rapidly progressing after HSCT (blue) versus non-transplanted patients (green) is shown in order to demonstrate the dynamic of disease progression between the two groups

All transplanted children with rapid disease progression after HSCT were already level 1 GMFC-MLD before transplantation. The interval between level 1 GMFC-MLD and level 2 GMFC-MLD was significantly different to the non-transplanted cohort (*p* = 0.02). One year after having entered level 1, all transplanted patients had entered level 2 of GMFC-MLD, and only 25% of the non-transplanted patients had entered level 2. Five out of the 35 non-transplanted patients were still level 1 at the end of observation period (Fig. 3).

### Prognostic values of baseline parameters for early outcome (Table 2)

Gross motor function at the time of HSCT was highly relevant to disease progression. Disease stabilization occurred in patients with level 0 GMFC-MLD (*p* = 0.013, *n* = 5) and a GMFM-88 of 100% (*p* = 0.003, *n* = 6).

**Table 2:** Predictive values for disease progression after HSCT. All *p* values were considered as descriptive

**GMFM < 100%**	***p*** **= 0.003**
**GMFC.MLD > 0**	***p*** **= 0.013**
FSIQ < 85	*p* = 0.079
**MR severity score > 17**	***p*** **= 0.003**
NCV < 40 m/s	*p* = 0.198
Age at onset	*p* = 0.690
Age at HSCT	*p* = 0.530

Regarding cognitive function and peripheral neuropathy, there was no difference between the outcome groups for FSIQ or the NCV at the time of transplantation. Mean baseline FISQ was 94.6 (range 68–114) for the group with stable disease and 82.8 (range 66–103) for the group with progression. At the time of HSCT, five patients exhibited a normal NCV (stable group *n* = 4, progressive group *n* = 1) and seven patients exhibited a pathological NCV (stable group *n* = 3, progressive group *n* = 4).

For the MR severity score, the stable outcome group exhibited a mean baseline score of 12.3 (range 0–21) and the group with clinical disease progression an MRI baseline score of 19.8 (range 18–24). A total MR severity score of above 17 at the time of HSCT was associated with disease progression after HSCT (*p* = 0.003).

Age of onset was not different between the 5 patients that had rapid progression after HSCT (mean age of onset 8.8 years, range 4.3–13.1) and the group with stable disease after HSCT (mean 7.6 years, range 4.6–9.8). Mean age at HSCT was 12.0 (range = 4.9–18.2) in the stable outcome group (*n* = 7) and 10.9 years (range = 5.2–13.9) in the progressive outcome group (*n* = 5), also showing no statistically significant difference (Table 2).

## Discussion

While the long-term outcome of HSCT in children with juvenile MLD has already been investigated systematically in recent years [5, 12, 15–18, 30–32], early outcome has been reported for MRI characteristics [9, 19, 22, 33–35], but not with respect to functional clinical parameters. In this paper, we analyzed clinical, electrophysiological, and MRI outcome parameters over the first 2 years after HSCT in children with juvenile MLD. Our results demonstrate that the early course after HSCT determines the long-term outcome. If clinical disease progression occurred after HSCT, this happened in the first 12 to 18 months after transplantation. Thus, neurological deterioration becomes less likely if patients remain stable throughout this period. Our findings thereby corroborate previous studies that analyzed outcomes 2 years post-transplantation and found stability at 2 years after HSCT to be predictive for further stability [5, 8–10, 33].

We also found that the five transplanted patients, who exhibited rapid disease progression after HSCT, deteriorated on average more rapidly in their gross motor function than a comparable group of non-transplanted patients. This, and the temporal association with HSCT, could indicate that rapid progression in their disease course was triggered by HSCT. There is already some evidence for more rapid disease progression following HSCT [10, 33]. The underlying pathomechanisms can only be speculated on. A possible factor could be the transplantation itself, e.g., toxic effects of chemotherapy. Drug-related neurotoxic effects on motor and cognitive impairment have been reported and a patient who is close to the rapid regression phase could be more sensitive to these effects [36]. Furthermore, deterioration in nerve conduction velocity has been associated with long-term toxicity caused by chemotherapy [37], which may not only impact on the peripheral nerves, but also on myelin sheaths in the CNS [14]. In addition, inflammatory responses induced by HSCT may influence progression of the course of disease. Only recently, disease progression in MLD has been shown to be strongly influenced by damage to microglia cells [38]. Furthermore, our findings show that transplanted patients with disease progression differed significantly in one key aspect from patients who remained stable: their abnormal gross motor function at the time of HSCT–even if impairment was only mild. This means that children who are already suffering from a neuropathology that is causing disturbances in their gross motor function at the time of HSCT are probably more vulnerable to transplant-related (inflammatory/toxic) stress and this might accelerate disease progression.

There were no changes in neuropathy after HSCT, as measured by NCV. Some patients exhibited pathological values, and some did not. There was no influence on motor impairment or on outcome after HSCT. This is in line with other studies which demonstrated that, while HSCT had an impact on the CNS, the PNS remained relatively unchanged, or even deteriorated, while brain lesions improved [17, 30, 33, 39]. The fact that peripheral neuropathy might respond better to gene therapy because of the higher enzyme levels that are achieved has been discussed previously [39].

With regard to the children’s condition at the time of HSCT, patients who remained stable throughout the entire observation period exhibited no gross motor impairment (GMFC-MLD = 0, GMFM = 100%) and few MRI changes (MR severity score ≤ 17) at baseline. This underlines the importance of carrying out HSCT at a very early stage of the disease and corroborates previous recommendations for HSCT in pre-symptomatic and mildly symptomatic children with juvenile MLD [5, 12–14, 16, 17]. Age of onset (or early-/late-juvenile onset) and age at HSCT were not predictive of early outcome after HSCT. Our findings suggest that the patient’s clinical (and MRI) status at HSCT might be more important than age of onset, although an earlier study showed that age of onset below 4 years might have a negative impact on outcome [12], highlighting the potentially more rapid disease progression in early juvenile patients. Also, the conditioning regimen used in our study did not show a clear impact on outcome after HSCT. Although it has been suggested in mice, that busulfan allows better migration of donor cells into the brain than treosulfan-based conditioning [40], Thiotepa and Fludarabin, which treosulfan were combined, penetrate well into the CNS. Clearly, larger (multicenter) studies are needed to investigate the effect of the conditioning regimen for MLD, balancing beneficial effects for engraftment/outcome and lessened neurotoxicity [14], but might also help in better addressing outstanding issues, e.g., developing recommendations for inclusion and exclusion criteria for HSCT.

All recommendations for transplantation in juvenile MLD suggest that patients should either be pre-symptomatic or in the very early stages of disease but vary when it comes to specifying the latter. Peters et al. refer to good neuropsychological function and independence in activities in daily life [5] and Musolino et al. mention “minimally symptomatic” patients [14]. Van Rappard et al. suggest the ability to walk without support and an FSIQ of above 75 [18]. Van der Broek et al. add well-matched grafts, the absence of cerebral atrophy and a normal performance score as predictive parameters for higher overall survival [16]. Regarding brain abnormalities, Groeschel et al. find that an MR severity score of lower than 17 is associated with a better outcome [12], Martin et al. suggest a value lower than 5 (without including atrophy) [17] and van Rappard et al. a value lower than 15 [18] in their respective cohorts. A correlation between the interval from onset of first symptoms to HSCT with clinical outcome has also been suggested as a relevant factor [9, 16, 17]. Some of the patients studied here had already been investigated with respect to long-term outcome after HSCT [12]. Predictive factors for a good clinical outcome here were a GMFC-MLD of 0 or 1, FSIQ of at least 85, age at onset older than 4 years, as well as an MR severity score of lower than 17. Interestingly, FSIQ at the time of HSCT was not predictive of long-term outcome in the current study with a smaller cohort; however, with respect to short-term outcome, gross motor deterioration correlated with cognitive deterioration. Children with stable motor function did not deteriorate in cognitive function, irrespective of the level of their baseline FSIQ.

## Conclusions

Our data show that the first 12 to 18 months after transplantation are decisive for disease stabilization or progression. Good gross motor function and few MRI abnormalities indicate a high probability of disease stabilization. Deterioration in cognitive function in this cohort paralleled deterioration in gross motor function. Patients who exhibited rapid and severe disease progression after HSCT deteriorated more rapidly than non-transplanted patients, indicating a triggering effect of HSCT on disease progression. Taken together, for a good prognosis, our data underline the importance of transplantation at an early stage of the disease without clear gross motor symptoms and a low MRI score.

## Supplementary information

**Additional file 1.** Transplant-related characteristics of patients.

## Data Availability

All relevant data are within the paper. Individual subject’s values from the whole patient cohort may be made available upon request addressed to the corresponding author, pending the approval of the Institutional Review Board of the University of Tuebingen, Germany.

## Abbreviations

HSCT: Hematopoietic stem cell transplantation
MLD: Metachromatic leukodystrophy
ARSA: Arylsulfatase A
MUD: HLA-matched unrelated donors
MFD: HLA-matched family donors
BW: Body weight
GMFC-MLD: Gross Motor Function Classification for MLD
GMFM-88: Gross Motor Function Measure 88
NCV: Nerve conduction velocity
GvHD: Graft vs host disease
VOD: Veno-occlusive disease
SIRS: Systemic inflammatory response syndrome
MRI: Magnetic resonance imaging
ATG: Anti-thymocyte globulin
FSIQ: Full-scale intelligence quotient
