# Supplementary material for: Early clinical course after hematopoietic stem cell transplantation in children with juvenile metachromatic leukodystrophy
Source: Mol Cell Pediatr. 2020 Sep 3;7:12. doi: 10.1186/s40348-020-00103-7 (PMC7483683; doi:10.1186/s40348-020-00103-7)
Supplement: Supplementary file 1 — Additional file 1. Transplant-related characteristics of patients. [file 40348_2020_103_MOESM1_ESM.docx]

**Supplemenentary Material**: Transplant-related characteristics of patients

| **ID** | **Sex** | **Age at HSCT +year** | **Donor source** | **Conditioning regime with dosage** | **Donor chimerism** | **GvHD prophylaxis** | **Cell dose [TNC/kg]** | **ANC <500 μl [day]** | **Complications** |
| --- | --- | --- | --- | --- | --- | --- | --- | --- | --- |
| **1** | male | 13.9 2014 | MUD (10/10) BM | Fludarabin 5 x 30 mg/m^2^ Treosulfan 2 x 14 g/m^2^  ATG Fresenius 3 x 20 mg/kg Thiotepa 1 x 10 mg/kg | >95% | MTX CsA | CD34^+^ 5.19x10^6^ CD3^+^ 3693.76x10^4^ | 16 | - |
| **2** | male | 6.0 2009 | MUD (10/10) PBSC CD3/CD19 | Fludarabin 5 x 30 mg/m^2^ Treosulfan 3 x 14 g/m^2^ Thymoglobulin 10 mg/kg Thiotepa 1 x 10 mg/kg | >95% | MTX CsA | CD34^+^ 7.04x10^6^ CD3^+^ 5007.55x10^4^ | 11 | acute GvHD I° |
| **3** | male | 11.8 2015 | MUD (9/10) BM | Fludarabin 5 x 30 mg/m^2^ Treosulfan 3 x 14 g/m^2^ Thymoglobulin 10 mg/kg Thiotepa 1 x 10 mg/kg | >95% | MTX CsA l | CD34^+^ 4.78x10^6^ CD3^+^ 3376.49x10^4^ | 14 | acute GvHD I° |
| **4** | female | 11.6 2014 | MMUD (8/10) PBSC | Fludarabin 5 x 30 mg/m^2^ Treosulfan 3 x 14 g/m^2^  ATG Fresenius 3 x 15 mg/kg Thiotepa 1 x 10mg/kg | >95% | MTX CsA Mycophenolatmofetil | CD34^+^ 22.32x10^6^ CD3^+^  2495.06x10^4^ | 10 | acute GvHD I° |
| **5** | female | 4.9 2001 | MUD (10/10) PBSC | Busulfan 4 x 3.2 mg/kg Cyclophosphamid 4 x 50 mg/kg ATG Fresenius 3 x 10 mg/kg | >95% | MTX CsA | CD34^+^ 10.4x10^6^ CD3^+^ 420.0x10^4^ | 12 | VOD |
| **6** | female | 5.2 2015 | MUD (10/10) BM | Fludarabin 5 x 30 mg/m^2^ Treosulfan 3 x 14 g/m^2^ Thymoglobulin 10 mg/kg Thiotepa 1 x 10 mg/kg | >95% | MTX CsA | CD34^+^ 3.43x10^6^  CD3^+^ 5081.31x10^4^ | 15 | Viremia |
| **7** | female | 13.7 2008 | MUD (10/10) BM | Busulfan 4 x 3.2 mg/kg Cyclophosphamid 2 x 60 mg/kg Thymoglobulin 10 mg/kg | >95% | MTX CsA | CD34^+^ 1.76x10^6^ CD3^+^ 1061.64x10^4^ | 18 | - |
| **8** | female | 15.2 2006 | MUD (10/10) BM | Busulfan 4 x 3.2 mg/kg Cyclophosphamid 2 x 60 mg/kg Thymoglobulin 10 mg/kg | >95% | MTX CsA | CD34^+^ 5.18x10^6^ CD3^+^ 3553.91x10^4^ | 15 | - |
| **9** | female | 18.2 2010 | MFD (10/10) BM | Fludarabin 5 x 30 mg/m^2^ Treosulfan 3 x 14 g/m^2^ Thymoglobulin 10 mg/kg Thiotepa 1 x 10mg/kg | >95% | MTX CsA | CD34^+^ 3.05x10^6^ CD3^+^ 2184.54x10^4^ | 14 | acute GvHD I° |
| **10** | male | 14.8 2012 | MUD (10/10) BM | Fludarabin 5 x 30 mg/m^2^ Treosulfan 3 x 14 g/m^2^ Thymoglobulin 10 mg/kg Thiotepa 1 x 10mg/kg | >95% | MTX CsA | CD34^+^ 2.29x10^6^ CD3^+^ 3165.57x10^4^ | 15 | - |
| **11** | female | 13.6 2015 | MFD (10/10) BM | Fludarabin 5 x 30 mg/m^2^ Treosulfan 3 x 14 g/m^2^ Thymoglobulin 10 mg/kg Thiotepa 1 x 10 mg/kg | >95% | Csa Tacrolimus | CD34^+^ 2.85x10^6^ CD3^+^ 3863.48x10^4^ | 11 | - |
| **12** | female | 10.1 2009 | MFD (10/10) BM | Fludarabin 5 x 30 mg/m^2^ Treosulfan 3 x 14 g/m^2^  Thymoglobulin 10 mg/kg | >95% | CsA | CD34^+^ 7.08x10^6^  CD3^+^ 3142.51x10^4^ | 10 | acute GvHD I° |
